# Supplementary material for: Tuft dendrite spikes are accompanied by selective input from distinct functional networks
Source: bioRxiv. 2026 Jun 11:2026.06.10.731464. Preprint. [Version 1] doi: 10.64898/2026.06.10.731464 (PMC13277889; doi:10.64898/2026.06.10.731464)
Supplement: Supplementary file 1 [file NIHPP2026.06.10.731464v1-supplement-1.pdf]

bioRxiv preprint doi: https://doi.org/10.64898/2026.06.10.731464; this version posted June 11, 2026. The copyright holder for this preprint (which was not certified by peer review) is the author/funder, who has granted bioRxiv a license to display the preprint in perpetuity. It is made available under a CC-BY 4.0 International license.

Supplementary Figures

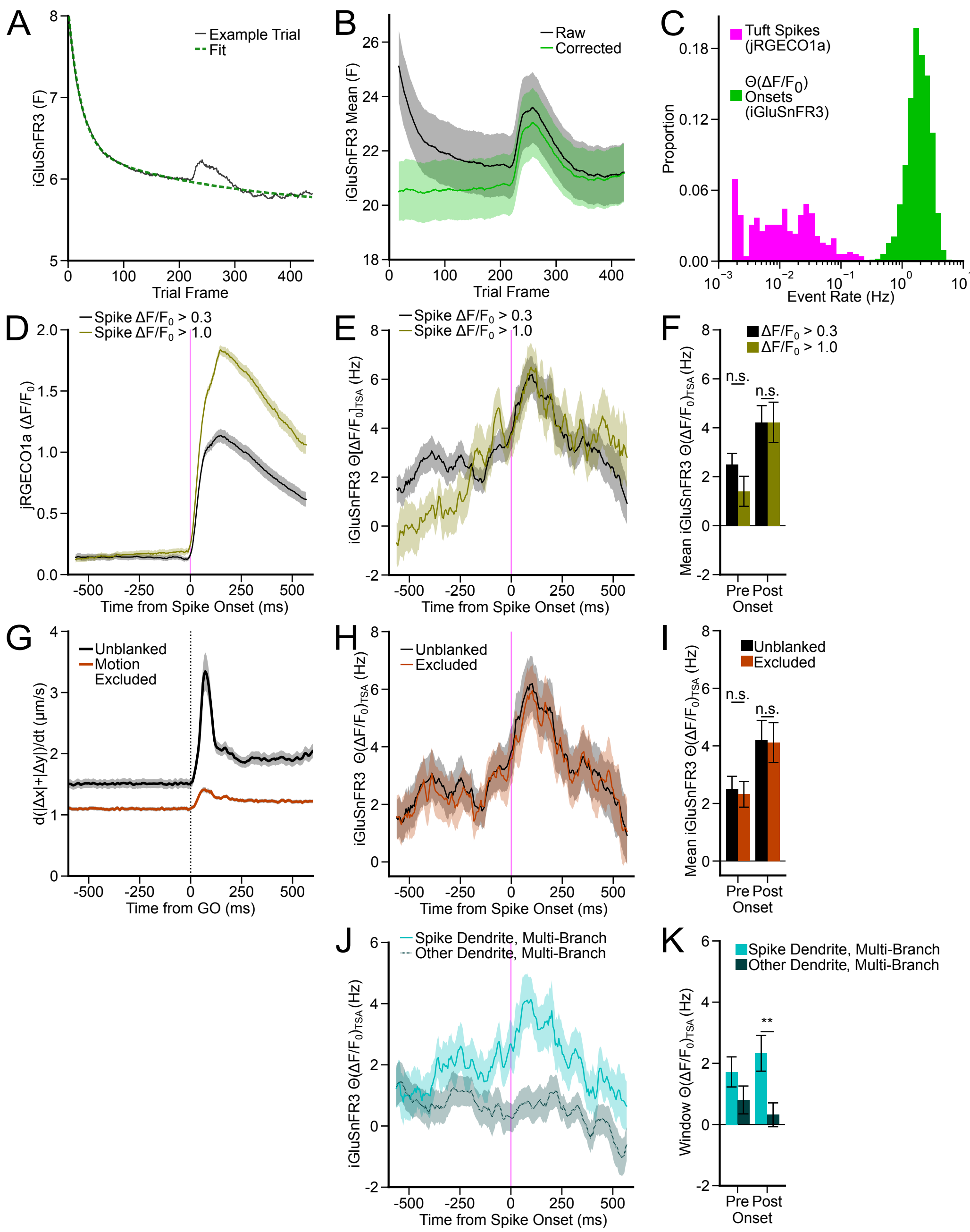

Fig. S1. Activity timecourse processing, rates, and robustness. ***A,*** Intra-trial photobleaching for example session. Whole-image trace (black) and double exponential decay model fit (dashed green; $\tau_{fast} = 23.0\,ms, \tau_{slow} = 287\,ms$; $R^2 = 0.99$). ***B,*** Mean of all trial averaged time course of synapses (no pre-processing) with (blue) and without (green) bleach correction for session in (A) ($n = 64$ synapses). ***C,*** Event rates of tuft spikes and synaptic $\Theta(\Delta F/F_0)$ events ($n = 313$ dendrites; $n = 2072$ synapses). ***D,*** Mean Ca^{2+} event transient across dendrites using a low ($0.3\,\Delta F/F_0$; olive; $n = 313$) and high ($1\,\Delta F/F_0$; gray; $n = 194$) minimum event amplitude threshold. ***E,*** Same as (D) except mean synaptic activity aligned to calcium transients ($\Theta(\Delta F/F_0)_{TSA}$; low $n = 826$; high $n = 333$). ***F,*** Mean pre-onset (-575–0 ms) and post-onset (0–575 ms) activity for data in (E). ***G,*** Mean inferred motion (black) and after largest motion frames were removed (dark red; $n = 52$ sessions). ***H,*** Mean synaptic $\Theta(\Delta F/F_0)$ activity aligned to tuft spikes before (black) and after (dark red) removing largest motion frames ($n = 826$ synapses). ***I,*** Mean pre-onset (-575–0 ms) and post-onset (0–575 ms) activity for data in (H). ***J,*** Average mean-subtracted $\Theta(\Delta F/F_0)_{TSA}$ activity aligned to putative multi-branch tuft spikes. SD synapses (cyan; $n = 485$). OD synapses (gray; $n = 1332$). ***K,*** Mean pre-onset (-575–0 ms) and post-onset (0–575 ms) activity for data shown in (J). Error shading and error bars are hierarchical bootstrap SEM. Statistical tests were two-tailed hierarchical difference tests (see Table S6)

bioRxiv preprint doi: https://doi.org/10.64898/2026.06.10.731464; this version posted June 11, 2026. The copyright holder for this preprint (which was not certified by peer review) is the author/funder, who has granted bioRxiv a license to display the preprint in perpetuity. It is made available under a CC-BY 4.0 International license.

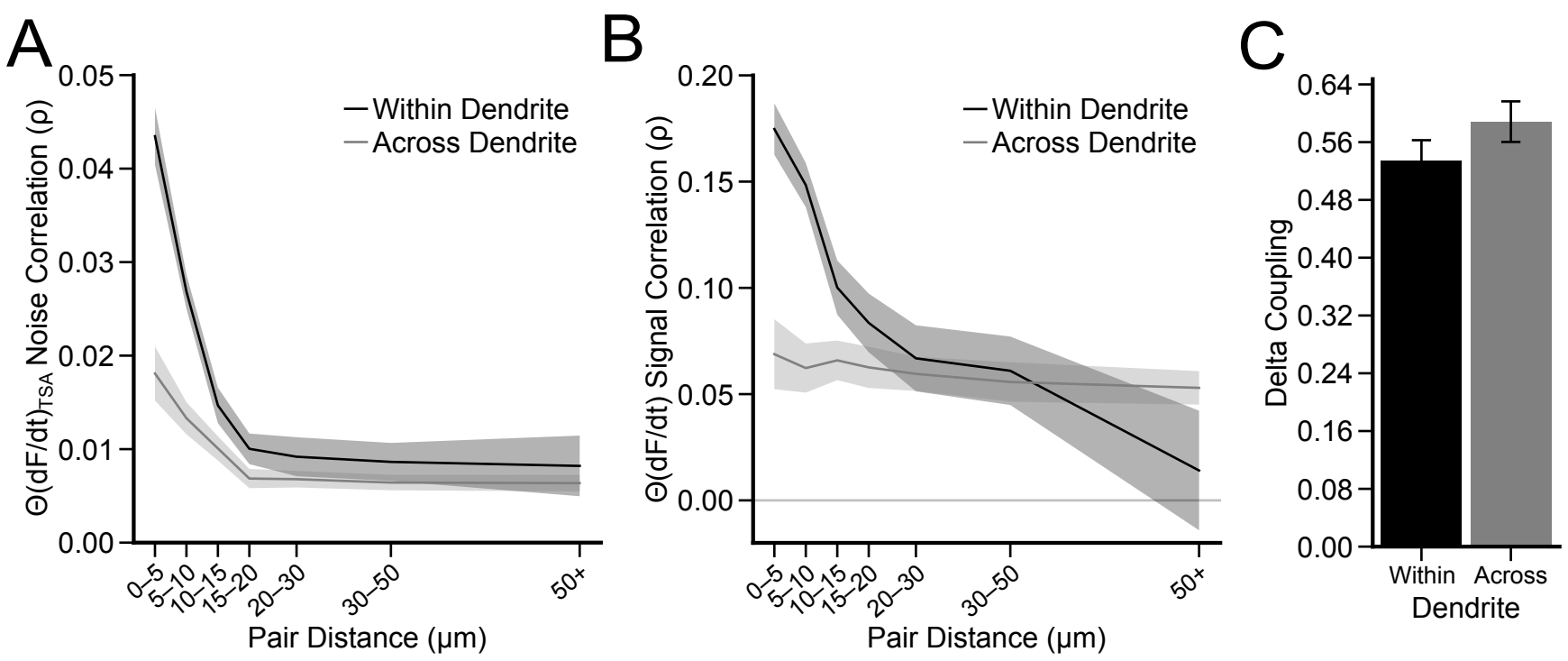

Fig. S2. Functional synaptic clustering analysis. ***A,*** Mean pairwise synaptic noise correlations (ρ) as a function of branch or euclidean distance ($\Theta(dF/dt)_{TSA}$) Pairs located on the same dendrite (black; $n = 6674$ pairs; $R^2 = 0.99$) or on different dendrites (gray; $n = 47775$ pairs; $R^2 = 0.98$). ***B,*** Same as (A), except for signal correlation ($\Theta(dF/dt)$ for within dendrite pairs (black; $R^2 = 0.96$) and across dendrite pairs (gray; $R^2 = 0.76$). ***C,*** Mean pairwise synaptic population coupling similarity for synapses located on the same or different dendrites ($n = 461$ synapses; two-tail hierarchical bootstrap mean difference test $p = 0.19$). Error shading and error bars: hierarchical bootstrap SEM.

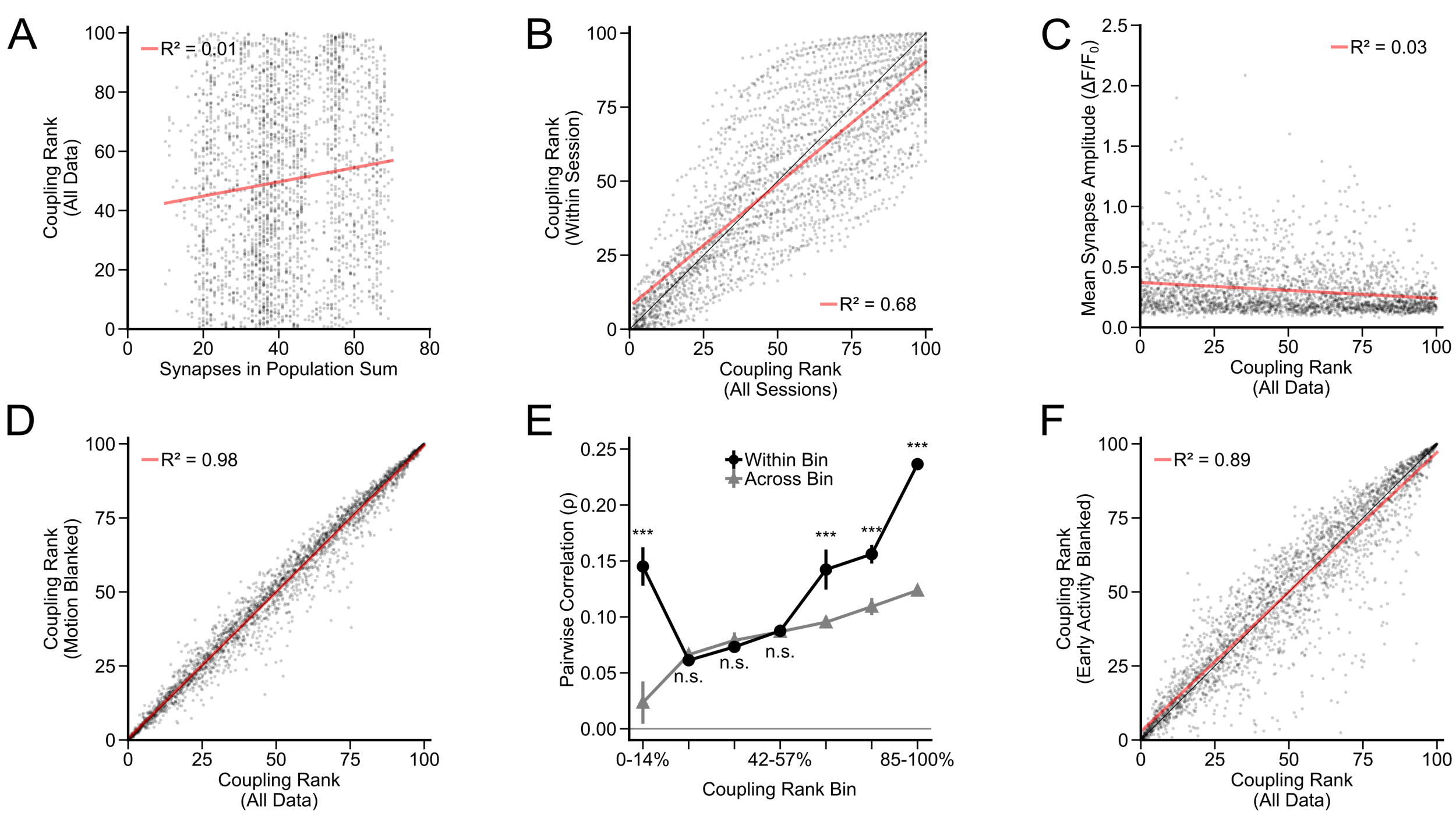

Fig. S3. Robustness of population coupling estimates and organization. ***A,*** Relationship between coupling rank and population size used to calculate rank. ($r = 0.12$; $n = 2591$ synapses). ***B,*** Relationship between synaptic coupling ranks when combining all sessions together versus ranks calculated using single sessions. Red line indicates linear fit ($r = 0.83$; $n = 2591$ synapses) ***C,*** Relationship between coupling rank and average individual $\Delta F/F_0$ transient size ($r = -0.18$; $n = 2591$ synapses). ***D,*** Relationship between synaptic coupling ranks before and after removing the top 15% of inferred high-motion frames (see Methods for further details). Red line indicates linear fit ($r = 0.99$; $n = 2591$ synapses). ***E,*** Mean correlation of synapse pairs by population coupling similarity during spontaneous behavior using $\Delta F/F_0$ traces including pairs in the same coupling range (black circles; $n = 1965$ pairs) and different coupling ranges (gray triangles; $n = 22424$ pairs). Error bars: hierarchical bootstrap SEM. Statistical significance: one-tail hierarchical bootstrap mean difference test (see Table S7). ***F,*** Relationship between coupling rank before and after removal of the early response period (0–0.5 s from GO cue) from analysis. Red line indicates linear fit ($r = 0.94$; $n = 2591$ synapses).

bioRxiv preprint doi: https://doi.org/10.64898/2026.06.10.731464; this version posted June 11, 2026. The copyright holder for this preprint (which was not certified by peer review) is the author/funder, who has granted bioRxiv a license to display the preprint in perpetuity. It is made available under a CC-BY 4.0 International license.

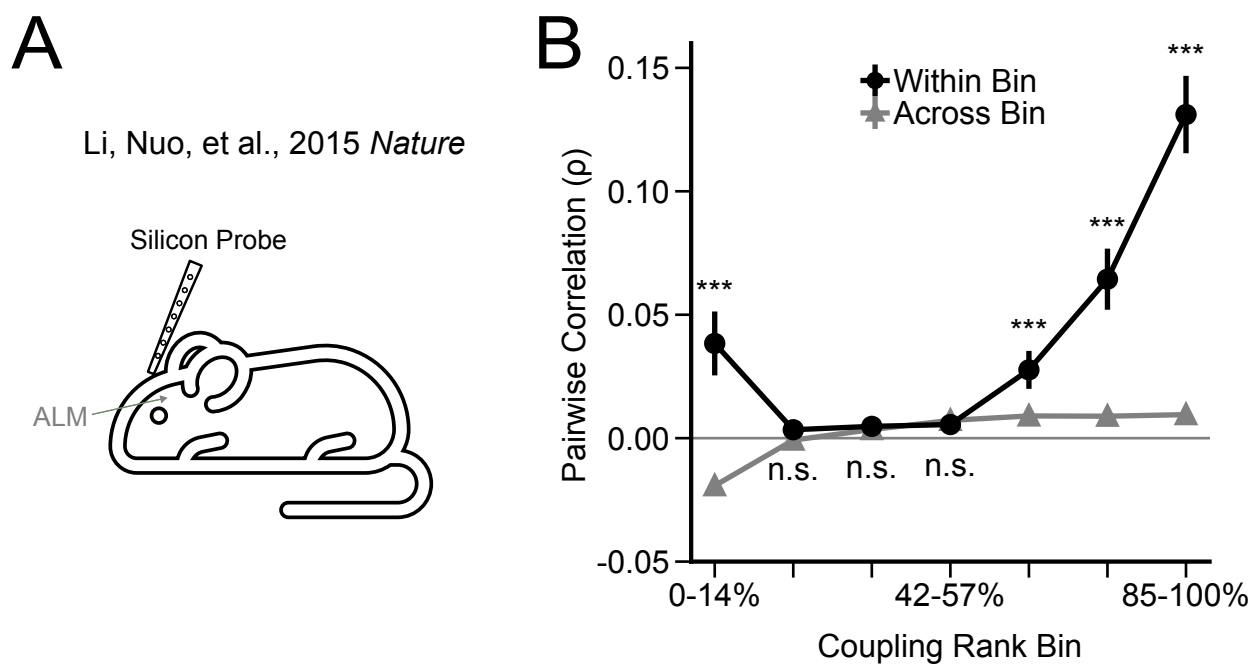

Fig. S4. Similar organization of population coupling in silicon probe recordings. ***A,*** Secondary data analysis measuring spiking activity at single neuron resolution in ALM using silicon probes in a similar behavior task (49). ***B,*** Spiking activity noise correlation (ρ) as a function of coupling similarity. Black circles represent pairs in the same coupling range ($n = 753$ pairs). Gray triangles represent pairs in different coupling ranges with one neuron belonging in a given range ($n = 6586$ pairs). Error bars: hierarchical bootstrap SEM. Statistical significance: one-tail hierarchical bootstrap mean difference test (see Table S8).

bioRxiv preprint doi: https://doi.org/10.64898/2026.06.10.731464; this version posted June 11, 2026. The copyright holder for this preprint (which was not certified by peer review) is the author/funder, who has granted bioRxiv a license to display the preprint in perpetuity. It is made available under a CC-BY 4.0 International license.

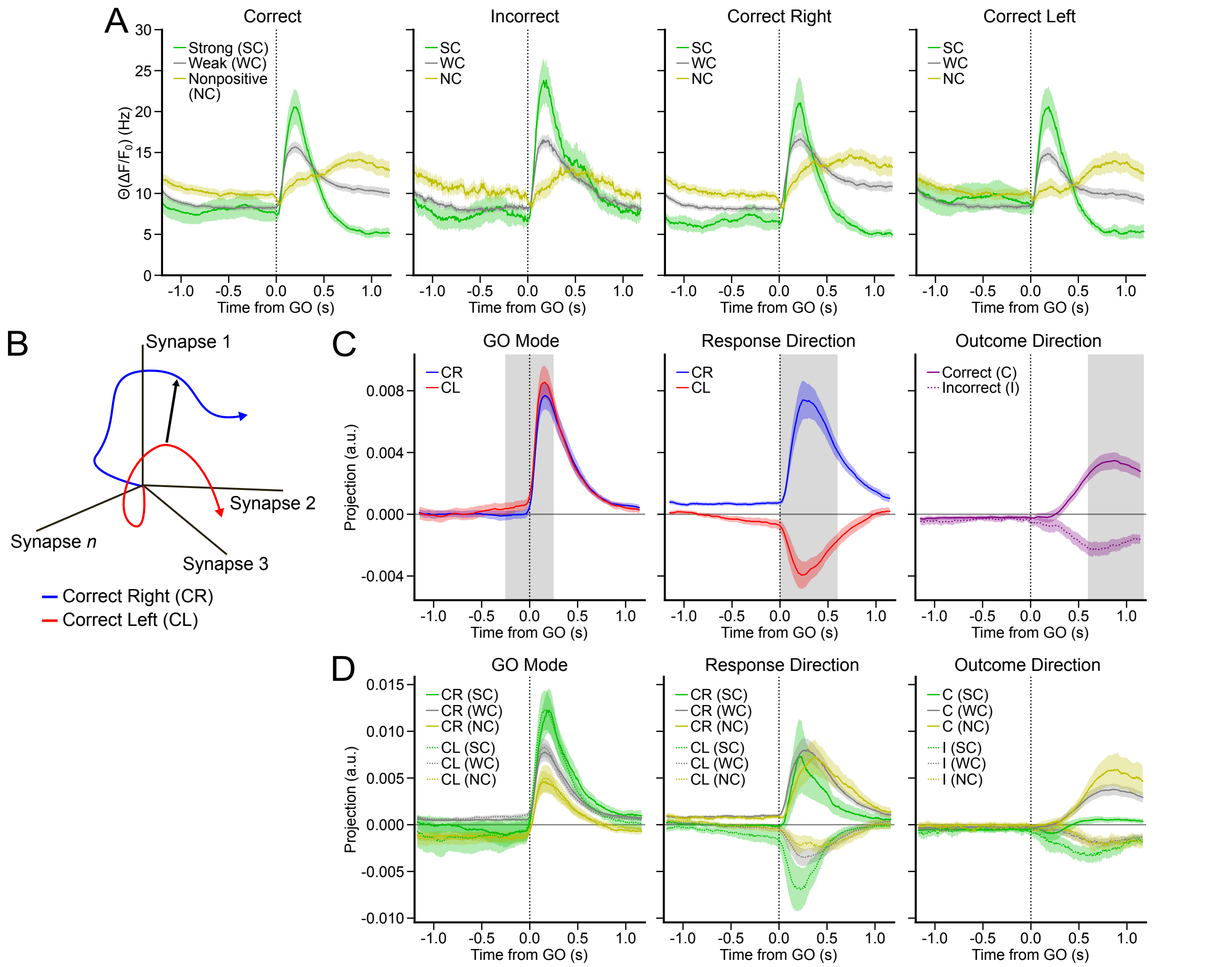

Fig. S5. Additional characterization of task coding and population coupling groups. ***A,*** Trial averaged mean $\Theta(\Delta F/F_0)$ aligned to GO cue (dotted black line) for correct (C), incorrect (I), correct right (CR), and correct left (CL) trial conditions for strong (SC), weak (WC) and nonpositive (NC) coupling groups (C/CR/CL *n*: $SC = 518$, $WC = 1555$, $NC = 518$ synapses; I *n*: $SC = 479$, $WC = 1267$, $NC = 434$ synapses). ***B,*** Visual depiction of activity mode calculation. ***C,*** Population mean for GO mode, Response and Outcome Direction activity for synapses. $\Theta(\Delta F/F_0)$ traces used for calculation. Gray shading indicates the epoch used to calculate activity-mode weights. GO Mode (-0.25–0.25 s) projected onto CR and CL trials. Response Direction (0–0.6 s) projected onto CR and CL trials. Outcome Direction (0.6–1.2 s) projected onto correct and error trials (GO Mode and Response Direction $n = 2591$ synapses; Outcome Direction $n = 2180$ synapses). ***D,*** Same as (C), except split up by population coupling group (GO Mode and Response Direction *n*: $SC = 518$, $WC = 1555$, $NC = 518$; Outcome Direction *n*: $SC = 479$, $WC = 1267$, $NC = 434$ synapses). Error shading: hierarchical bootstrap SEM.

bioRxiv preprint doi: https://doi.org/10.64898/2026.06.10.731464; this version posted June 11, 2026. The copyright holder for this preprint (which was not certified by peer review) is the author/funder, who has granted bioRxiv a license to display the preprint in perpetuity. It is made available under a CC-BY 4.0 International license.

Tables

Table S1. Two-tailed hierarchical bootstrap mean difference tests for population synaptic activity aligned to tuft spikes (Fig. 2).

Comparison	Mean difference (Hz)	p value
$\Theta(\Delta F/F_0)$ (Fig. 2F)		
SD_{pre} vs OD_{pre}	2.5	0.002
SD_{post} vs OD_{post}	5.2	6.0×10^{-4}
SD_{post} vs SD_{pre}	2.2	0.009
$\Theta(\Delta F/F_0)_{TSA}$ (Fig. 2J)		
SD_{pre} vs OD_{pre}	1.87	6.0×10^{-4}
SD_{post} vs OD_{post}	3.75	6.0×10^{-4}
SD_{post} vs SD_{pre}	1.72	0.003
$\Theta(dF/dt)_{TSA}$ (Fig. 2N)		
SD_{pre} vs OD_{pre}	1.15	6.0×10^{-4}
SD_{post} vs OD_{post}	1.33	6.0×10^{-4}
SD_{pre} vs SD_{post}	0.08	0.75

p values for each of the three measures separately corrected using Holm Method. SD n = 826 synapses. OD n = 1922 synapses.

Table S2. One-tailed hierarchical bootstrap mean difference tests for same and different coupling range pairwise noise correlations (Fig. 3D).

Bin	Mean difference (ρ)	p value
0.00%–14.29%	0.02	7.0×10^{-3}
14.29%–28.57%	0.002	0.11
28.57%–42.86%	0.0007	0.24
42.86%–57.14%	0.002	0.11
57.14%–71.43%	0.01	7.0×10^{-3}
71.43%–85.71%	0.02	7.0×10^{-3}
85.71%–100.00%	0.06	7.0×10^{-3}

p values corrected using Holm method. Same coupling range $n = 9561$ pairs. Different coupling range $n = 71212$ pairs.

Table S3. Two-tailed hierarchical bootstrap mean difference tests for synaptic activity aligned to tuft spikes by coupling group (Fig. 3F).

Comparison	Mean difference (Hz)	p value
SC_{SD} vs WC_{SD}	1.1	0.002
SC_{SD} vs NC_{SD}	1.0	0.02
NC_{SD} vs WC_{SD}	0.08	0.7
SC_{SD} vs SC_{OD}	1.96	6.0×10^{-4}
WC_{SD} vs WC_{OD}	0.59	6.0×10^{-4}
NC_{SD} vs NC_{OD}	0.77	6.0×10^{-4}

Between-coupler-group SD, and SD vs OD p values separately corrected using Holm method. NC_{SD} $n = 160$ synapses, NC_{OD} $n = 384$, WC_{SD} $n = 522$, WC_{OD} $n = 1156$, SC_{SD} $n = 144$, SC_{OD} $n = 382$.

bioRxiv preprint doi: https://doi.org/10.64898/2026.06.10.731464; this version posted June 11, 2026. The copyright holder for this preprint (which was not certified by peer review) is the author/funder, who has granted bioRxiv a license to display the preprint in perpetuity. It is made available under a CC-BY 4.0 International license.

Table S4. Two-tailed hierarchical difference tests for synaptic activity modes aligned to GO cue. (Fig. 4H).

Comparison	Mean difference (a.u.)	p value
GO Mode		
SC vs WC	0.0035	0.07
SC vs NC	0.0060	0.002
WC vs NC	0.0026	0.07
Response Direction		
SC vs WC	0.0000	1.0
SC vs NC	0.0008	0.8
WC vs NC	0.0008	0.6
Outcome Direction		
NC vs SC	0.0020	6.0×10^{-4}
WC vs SC	0.0010	6.0×10^{-4}
NC vs WC	0.0009	0.3

p values for each activity mode separately corrected using Holm method. GO Mode and Outcome Direction synapse n: $NC = 434$, $WC = 1267$, $SC = 479$; Response Direction synapse n: $NC = 518$, $WC = 1555$, $SC = 518$.

Table S5. One-tailed hierarchical difference tests for GO Mode and Outcome Direction synaptic activity aligned to tuft spikes (Fig. 5).

Comparison	Mean difference (a.u.)	p value
GO Mode (Fig. 5B)		
SC_{SD} vs WC_{SD}	0.003	0.1
SC_{SD} vs NC_{SD}	0.005	0.003
WC_{SD} vs NC_{SD}	0.002	0.007
SC_{SD} vs SC_{OD}	0.004	0.02
WC_{SD} vs WC_{OD}	0.002	0.01
NC_{SD} vs NC_{OD}	0.0006	0.02
Outcome Direction (Fig. 5D)		
NC_{SD} vs SC_{SD}	0.001	0.02
NC_{SD} vs WC_{SD}	0.001	0.02
WC_{SD} vs SC_{SD}	0	0.6
NC_{SD} vs NC_{OD}	0.001	0.05
WC_{SD} vs WC_{OD}	-0.0003	1
SC_{SD} vs SC_{OD}	-0.0002	1

p values separately corrected using Holm method for each group of hypotheses (GO Mode SD across coupler groups, GO Mode SD vs OD, Outcome Direction SD across coupler groups, Outcome Direction SD vs OD). GO Mode synapse n: $SC_{SD} = 90$, $WC_{SD} = 287$, $NC_{SD} = 98$, $SC_{OD} = 355$, $WC_{OD} = 1040$, $NC_{OD} = 353$. Outcome Direction synapse n: $SC_{SD} = 83$, $WC_{SD} = 269$, $NC_{SD} = 96$, $SC_{OD} = 328$, $WC_{OD} = 1000$, $NC_{OD} = 342$.

bioRxiv preprint doi: https://doi.org/10.64898/2026.06.10.731464; this version posted June 11, 2026. The copyright holder for this preprint (which was not certified by peer review) is the author/funder, who has granted bioRxiv a license to display the preprint in perpetuity. It is made available under a CC-BY 4.0 International license.

Table S6. Two-tailed hierarchical bootstrap mean difference tests for tuft spike-associated synaptic activity control analyses (Fig. S1).

Comparison	Mean difference (Hz)	p value
Event-threshold comparison (Fig. S1F)		
Low thresh.$_{pre}$ vs high thresh.$_{pre}$	1.0	0.3
Low thresh.$_{post}$ vs high thresh.$_{post}$	0.01	1
Motion-removal control (Fig. S1I)		
Control$_{pre}$ vs motion removed$_{pre}$	0.2	1
Control$_{post}$ vs motion removed$_{post}$	0.08	1
Multi-branch tuft spikes (Fig. S1K)		
SD$_{pre}$ vs OD$_{pre}$	0.9	0.16
SD$_{post}$ vs OD$_{post}$	2.0	0.004

p values separately corrected using Holm method for each group of hypotheses (tuft spike event threshold, motion control, multi-branch spikes). Low threshold synapse $n = 826$ High threshold synapse $n = 333$. Motion (both groups) synapse $n = 826$. Multi-branch: SD synapse $n = 485$, vs OD synapse $n = 1332$.

Table S7. One-tailed hierarchical mean difference tests for same and different coupling range pairwise correlations during spontaneous behavior (Fig. S3E).

Bin	Mean difference (ρ)	p value
0.00%–14.29%	0.1	7.0×10^{-4}
14.29%–28.57%	-0.005	1
28.57%–42.86%	-0.006	1
42.86%–57.14%	0.0007	1
57.14%–71.43%	0.05	7.0×10^{-4}
71.43%–85.71%	0.05	7.0×10^{-4}
85.71%–100.00%	0.1	7.0×10^{-4}

p values corrected using Holm method. Mice $n = 1$. Session $n = 3$. Same coupling range $n = 1965$ pairs. Different coupling range $n = 22424$ pairs.

Table S8. One-tailed hierarchical mean difference tests for same and different coupling range pairwise spiking correlations at somas in ALM (Fig. S4B).

Bin	Mean difference (ρ)	p value
0.00%–14.29%	0.06	7.0×10^{-4}
14.29%–28.57%	0.004	0.12
28.57%–42.86%	0.001	0.61
42.86%–57.14%	-0.002	0.76
57.14%–71.43%	0.02	7.0×10^{-4}
71.43%–85.71%	0.06	7.0×10^{-4}
85.71%–100.00%	0.1	7.0×10^{-4}

p values corrected using Holm method. Mice $n = 11$. Session $n = 20$. Same coupling range $n = 753$ neuron pairs. Different coupling range $n = 6586$ neuron pairs.
